# Data-driven normative values based on generative manifold learning for quantitative MRI

**DOI:** 10.1038/s41598-024-58141-4

**Published:** 2024-03-30

**Authors:** Arnaud Attyé, Félix Renard, Vanina Anglade, Alexandre Krainik, Philippe Kahane, Boris Mansencal, Pierrick Coupé, Fernando Calamante

**Affiliations:** 1 GeodAIsics, Biopolis, 38043 Grenoble, France; 2 Department of Neuroradiology and MRI, SFR RMN Neurosciences, University Grenoble Alpes Hospital, Grenoble, France; 3 Department of Neurology, University Grenoble Alpes Hospital, Grenoble, France; 4 CNRS, Univ. Bordeaux, Bordeaux INP, LABRI, UMR5800, 33400 Talence, France; 5 School of Biomedical Engineering, The University of Sydney, Sydney, NSW 2006 Australia; 6 Sydney Imaging-The University of Sydney, Sydney, Australia

**Keywords:** Diagnostic markers, Learning algorithms

## Abstract

In medicine, abnormalities in quantitative metrics such as the volume reduction of one brain region of an individual versus a control group are often provided as deviations from so-called normal values. These normative reference values are traditionally calculated based on the quantitative values from a control group, which can be adjusted for relevant clinical co-variables, such as age or sex. However, these average normative values do not take into account the globality of the available quantitative information. For example, quantitative analysis of T1-weighted magnetic resonance images based on anatomical structure segmentation frequently includes over 100 cerebral structures in the quantitative reports, and these tend to be analyzed separately. In this study, we propose a global approach to personalized normative values for each brain structure using an unsupervised Artificial Intelligence technique known as generative manifold learning. We test the potential benefit of these personalized normative values in comparison with the more traditional average normative values on a population of patients with drug-resistant epilepsy operated for focal cortical dysplasia, as well as on a supplementary healthy group and on patients with Alzheimer’s disease.

## Introduction

Automatic quantitative tools for Magnetic Resonance Imaging (MRI brain analysis are a very valuable resource for the diagnosis and management of neurologic diseases, particularly quantitative volume measurements based on automatic segmentation of brain structures including cortical subdivision, deep grey nuclei or hippocampi. Such analysis, based on segmentation of T1-weighted images, can now be reliably obtained using deep learning techniques^1–3^, which show domain shift robustness and high potential for generalization.

Once segmentation volumes are obtained, the detection of volume abnormalities in each structure is classically achieved based on normative lifespan data^4,5^ (i.e. the model of the developing trajectories/curves): individuals falling outside the normative curves are considered as harboring an abnormality. The largest and most inclusive dataset for reference standards was recently proposed with 123,984 MRI scans from 101,457 participants^4^. The main idea of proposing such a large reference cohort is to be robust to brain morphometrics variations, such as variation in scanner platforms, sequences and data quality.

These approaches are used in a number of popular software tools, such as VolBrain^6^, Qyscore^7^, Neuroquant^8^, or IcoBrain DM^9^. One potential limitation with these approaches is the use of civil age and sex as co-variables. For example, the available reference data are not always equally distributed across all ages leading to a high variance of reference cohorts in many cases. A framework allowing to better select appropriate controls from the reference cohort based on multiple quantitative measures of the brain, instead of just civil age, would allow to improve the modeling of non-Gaussian predictive distributions. In addition, there is a need to develop methods aiming at proposing personalized normative values to detect local cortical alterations while taking other cortical regions into account. This is not addressed by traditional methods, which compare each region *independently*.

Unsupervised manifold learning has long been used in medical data analysis, for example to decrease the noise and reconstruction artifacts in the pre-processing of medical imaging data^10^, for drug repurposing by clustering gene expression ^11^, or for single cell identification^12^. In this study, we exploit this type of machine learning methods to provide a personalized normative model while considering all structures in a more global approach.

The first step of manifold learning often involves a dimensionality reduction for high-dimensional data, which can be linear or non-linear using Riemannian approximation^13,14^. Manifold learning algorithms aim to represent these complex datasets within a more restricted space, while preserving the topology and the initial relative distance between each data characteristic in the original space. The dimension reduction step is not just a simple tool permitting the visualization of complex data, but it also allows to link the variables from the original space before the application of manageable statistical models. This type of approach was recently proposed to generate synthetic data within the reduced latent space using a model of generative manifold learning for the identification of white matter abnormalities based on diffusion MRI^15^. In that work, the *TractLearn* algorithm was proposed as a statistical learning workflow optimized for MRI diffusion data, allowing to model inter-individual variability and predict structural changes in patients with mild Traumatic Brain Injury. This effectively involves creating “digital twin”, i.e. new individual from the nearest set in the manifold space of healthy controls included in the learning database, and to use these digital twins to enable the personalization of normative values at each brain structure tested. The difference between the newly created digital twin and the tested individual allows a more sensitive detection of quantitative abnormalities.

Here we propose a combined manifold learning approach for quantitative normative modeling applications in medicine, involving the construction of a geodesic learning framework that we referred to as *GeoNorm,* based on a large cohort of healthy controls, allowing the estimation of personalized normative values for each cerebral structure *simultaneously*. We demonstrate the framework by mapping the brain abnormalities of patients with drug-resistant epilepsy in a cohort of patients who underwent corticectomy for type 2 focal cortical dysplasia (FCDII) for which the pathologist confirmed the presence of hypertrophy of the operated cortex. Our new technology based on generative manifold learning is compared with a traditional lifespan model providing average normative values. Furthermore, *GeoNorm* is also used in a supplementary cohort of controls to ensure that the proposed personalized normative values are not overestimating the presence of cortex abnormalities in comparison with the average normative values. Finally, the new method is also applied to patients with Alzheimer’s disease to demonstrate its capability on a different clinical application.

## Results

### Diagnostic performance in epileptic patients

**Figure 1:**
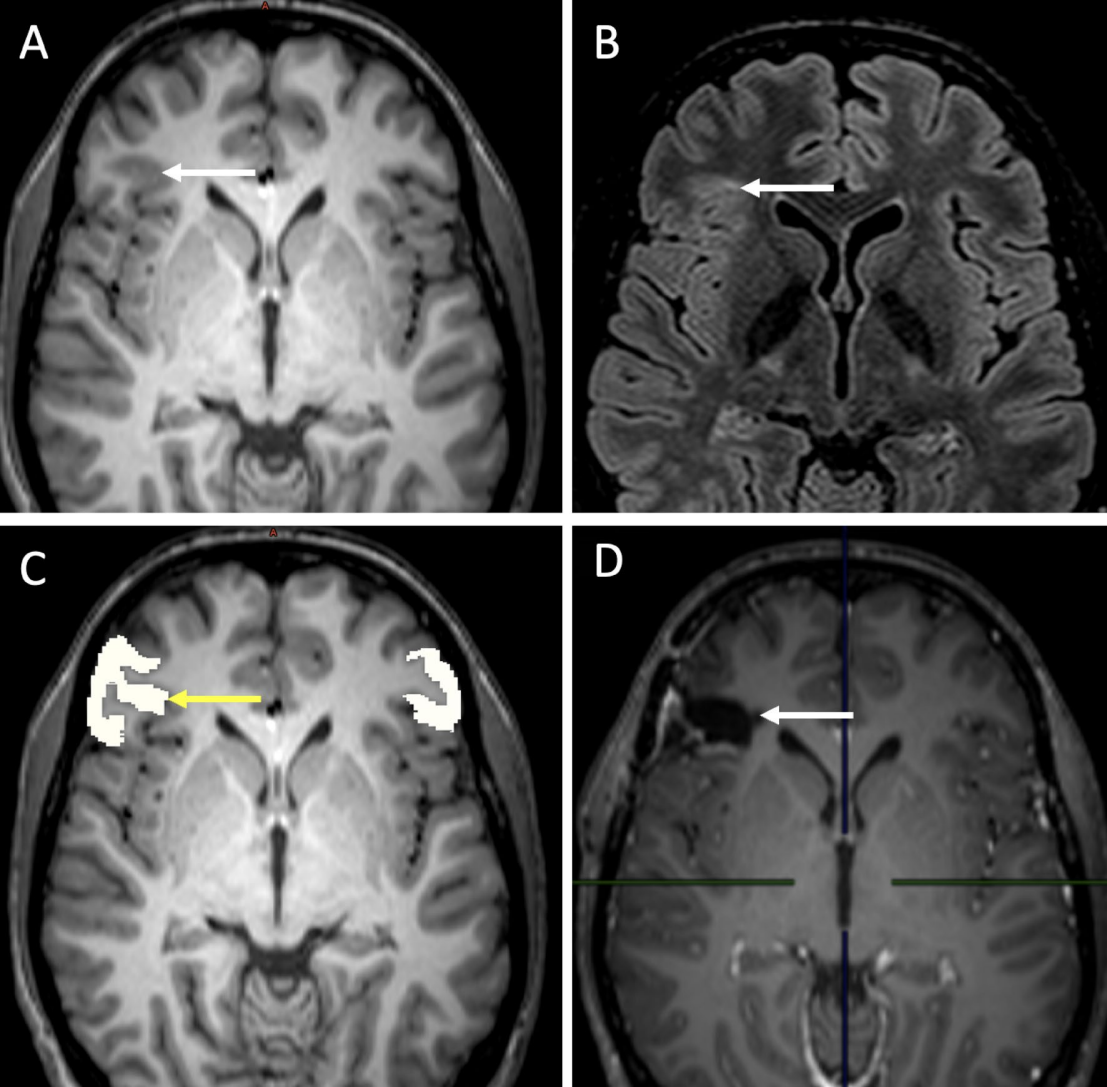
Example of a 18-year old patient with surgically confirmed FCDII of the right inferior triangular gyrus; MRI was considered abnormal by the senior radiologist. (**A**) Presurgical T1-weighted imaging: the white arrow shows focal thickening of the cerebral cortex at the level of the right inferior triangular gyrus. (**B**) Presurgical FLAIR sequence: the white arrow shows a subcortical region with FLAIR signal hyperintensity at the level of the dysplastic zone. (**C**) AssemblyNet segmentation of the right inferior triangular gyri, the yellow arrow shows the right sided region. (**D**) Postsurgical T1-weighted scan: postoperative cavity (white arrow).

All 28 epileptic patients considered in this study had an increase of the estimated cortical volume confirmed by post-operative tissue analysis. Among these, the average normative values from the LifeSpan model (i.e., the ‘traditional’ approach) identified 11 out of 28 patients with increase of estimated cortical volume, while our generative manifold learning analysis *GeoNorm* identified 17 out of 28 patients with increase of estimated cortical volume based on the personalized normative values (a further 55% more subjects detected). Figure 1 shows an example of a patient with confirmed FCDII. In this patient, AssemblyNet ^1^ estimated the inferior triangular gyrus occupies 0.35% of the total ICV. The LifeSpan average normative range for this part of the cortex was [Inferior: 0.21 Superior: 0.39]%, therefore considered normal. In contrast, the personalized normative values from GeoNorm found the following normative range: [0.21; 0.34]%, therefore classifying the right inferior triangular gyrus as abnormally increased.

The sensitivity of the personalized normative values on the resected zone was 0.67 (i.e. 17 patients out of 28 with cortical hypertrophy on MRI further confirmed by surgery) while that of the average normative values was 0.39 (i.e. 11 patients out of 28). Note that 0.67 was also the sensitivity of the radiological report (i.e. 17 patients out of 28, see Table 1).

**Table 1:** Relevant characteristics of the 28 epileptic patients included in the study.

Patient number	Sex	Age (years)	Age at epilepsy onset (years)	Surgery	Radiological report	Average normative values	Personalized normative values
1	F	10	0.6	R occipital	R occipital	Increased	Increased
2	M	10	4	L frontal	L frontal	Increased	Increased
3	F	11	1	R frontal	R frontal	NA	Increased
4	M	18	11	R frontal	R frontal	NA	Increased
5	M	19	4	R temporal	NA	NA	Increased
6	M	19	6	L frontal	L frontal	NA	NA
7	M	22	0.5	L frontal	L frontal	NA	Increased
8	F	23	5	L frontal	L frontal	Increased	Increased
9	M	26	3	R parietal	NA	Increased	Increased
10	M	29	4	R frontal	R frontal	NA	NA
11	F	29	9	R SMA	NA	NA	Increased
12	F	29	4	R frontal	R frontal	Increased	Increased
13	F	33	14	L SMA	NA	NA	NA
14	F	34	13	R frontal	NA	NA	NA
15	F	34	12	R parietal	NA	Increased	Increased
16	M	34	3	L frontal	L temporal	Increased	Increased
17	F	35	21	R temporal	R temporal	NA	NA
18	M	35	1	R temporal	R HS	Increased	Increased
19	M	40	25	L temporal	NA	NA	NA
20	M	40	7	R frontal	NA	NA	NA
21	F	41	28	R frontal	NA	NA	NA
22	M	42	15	L frontal	NA	NA	NA
23	M	42	18	L frontal	bilateral HS	Increased	Increased
24	M	44	4	R frontal	R frontal	NA	NA
25	M	47	4	L SMA	L SMA	NA	Increased
26	F	47	8	L frontal	L frontal	NA	NA
27	F	52	39	R temporal	R HS	Increased	Increased
28	M	57	22	L parietal	NA	Increased	Increased

The surgery column provided the gold standard for the dysplasia location. Average and Personalized normative values reported the potential presence of increase of estimated cortical volume (labelled increased) or the absence of reported abnormality (NA). The radiological report summarized the visual analysis of the radiologist, either by describing any abnormality from different MRI sequence (here reported with the location of the abnormality) or the absence of reported abnormality (NA).

M, male; F, female; R, right; L, left. SMA, supplementary motor area. HS, Hippocampal Sclerosis. NA, no abnormality.

Focusing on patients with pre-identified radiological abnormalities (n = 17), the average normative values found a total of 8 with increased estimated cortical volume, whereas personalized normative values identified 12 patients. In patients without radiologically described abnormalities (n = 11), average normative values found 3 cortical zones with hypertrophy and the personalized normative values found 5 in total, all consistent with the post-operative pathological analysis. The McNemar Test showed significant disagreement (p < 0.05) between the performance of the LifeSpan model and that of the generative manifold learning.

**Figure 2:**
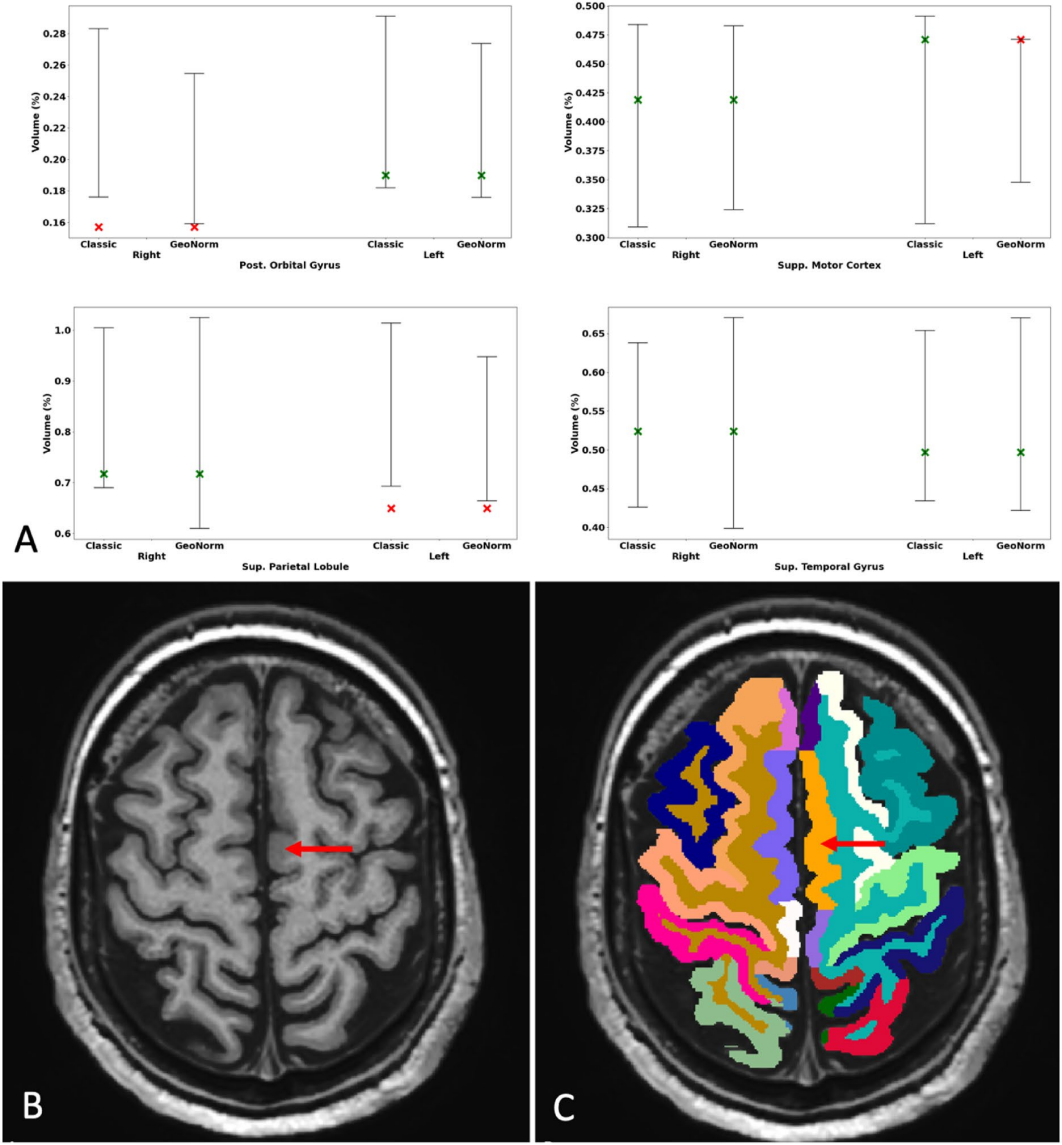
Normative range for the % volume of four structures for the 47-year-old randomly selected patient with FCD in the left supplementary motor cortex (**A**, Subject 25). The variation between normative ranges depends on the anatomical area. For example, the LifeSpan-based normative values placed the patient’s left supplementary motor cortex in the upper end of the range but still within the normal range (green cross), while GeoNorm considered this structure outside of the personalized normative values (red cross). Both models also found the left superior parietal lobule as atrophic, but this was less severe using GeoNorm. The Presurgical T1-weighted imaging (**B**) and AssemblyNet segmentation (**C**) are shown in the bottom row. For example, for the left supplementary motor cortex, AssemblyNet classified it as normal, while the GeoNorm personalized normative values classified it as with increased volume.

We randomly selected one patient (subject number 25 in Table 1), providing a comparison between some example areas and the corresponding MRI scan as shown in Fig. 2. For this patient, AssemblyNet estimated the left supplementary motor cortex as representing 0.47% of the volume of all the structures. The traditional average normative range for this part of the cortex was [0.31; 0.49], therefore considered as normal. The GeoNorm personalized normative values found the following normative range: [0.35; 0.47]%, therefore classifying the left supplementary motor cortex as with increased volume.

Furthermore, in this cohort, despite the lack of gold standard, we investigated the number of ROIs in the *non-operated* brain with cortical volume estimated as decreased or increased (Table 2).

**Table 2:** Mean number of cortical regions detected with either hypertrophy (increased) or atrophy + hypertrophy (Decreased + Increased) in the remaining non-operated 131 brain regions of the 28 epileptic subjects.

	Average normative values (LifeSpan)	Personalized normative values (GeoNorm)
Decreased + Increased	14 ± 6 (0.03% ± 0.015)	9 ± 4 (0.024% ± 0.01)
Increased	10 ± 6 (0.028% ± 0.015)	6 ± 4 (0.016% ± 0.01)

Percentages represent proportion of the total of cortical regions with abnormalities.

### Diagnostic performance in the supplementary healthy cohort

Table 3 shows the number of ROIs with decreased and increased volume in cortical regions. The Wilcoxon signed rank test was statistically significant when combining decreased and increased cortical regions (statistic = 7.5; p-value = 4.45 × 10^–5^), yet not for increased regions alone (statistic = 172.5; p-value = 0.48).

**Table 3:** Mean number of cortical regions detected with either hypertrophy (Increased) or atrophy and hypertrophy (Decreased + Increased) in the 132 brain regions of the second control dataset (n = 30).

	Average normative values (LifeSpan)	Personalized normative values (GeoNorm)
Decreased + Increased	11 ± 5 (0.032% ± 0.014)	7 ± 3 (0.02% ± 0.008)
Increased	5 ± 3 (0.014% ± 0.008)	5 ± 3 (0.014% ± 0.008)

Percentages represent proportion of the total of cortical regions with abnormalities.

### Performance in the Alzheimer’s disease cohort

As the choice of a single disease model raises questions for generalization, we also applied our proposed framework to the ADNI multicenter database for Alzheimer’s Disease (AD), both for AD patients and controls. We targeted the hippocampi of patients as a common atrophic structure in these patients, despite a small subset of patients described without apparent radiological atrophy ^26^. Personalized normative values significantly detected more hippocampus atrophy in comparison with average normative values in the pMCI group and in the AD group. There was no significant difference in the control group (supplementary table 1).

## Discussion

We have introduced *GeoNorm* as a new framework for normative analysis of individual subjects based on manifold learning. We showed that the generative manifold model for data-driven personalized normative values provided a better detection of focal cortex hypertrophy in comparison with the classic LifeSpan model providing average normative values. Importantly, these epilepsy patients had been specifically selected as they all had confirmed post-surgical histology analysis: all detected increased estimated cortical volume were confirmed by pathological analysis. In addition, personalized normative values detected fewer regions falling outside the normative curves in the other non-operated regions of epileptic brains, as well as in a second cohort of 30 healthy controls.

A major difference between the generative manifold learning model and the traditional lifespan models is the use of age as a co-variable for the latter. By comparing epileptic patients with digital twins built from the closest (in the manifold space) set of brain quantitative values from the healthy control group independently from age, we better capture the heterogeneity of aging in the general ‘normal’ population. In other words, the selected closest 30 normal controls are chosen with a global perspective on all 132 cortical metrics, which should better reflect the ‘*brain age’*. It is based on the assumption that the 30 selected controls, based on the 132 quantitative values coming from AssemblyNet reports, allow to more precisely model non-Gaussian predictive distributions and, thus, detect subtler local quantitative variations. Over the lifespan, the inter-subject variability of the volume of each anatomical structure tends to increase either due to environmental or genetic factors. The quantitative pairing using the nearest subjects from a more global data viewpoint (rather than comparing with a Euclidean mean—see a toy example in Supplementary Figs. 1 and 2) allows the improved detection of atypical anatomical regions at the subject level.

The generation of synthetic data is more common in machine learning concerning generative adversarial networks^16^ in which a convolutional neural network synthesizes artificial images and another network tests them. While normally used to refer to a virtual clone of an image or device, a ‘digital twin’ in our study refers to a series of quantitative measurements that can be considered as a suitable representation of the *reference* brain measures for comparing to a specific patient. The manifold learning space could be either estimated in a linear or non-linear manner for the generation of synthetic data^14^. Principal components analysis represents the typical example of linear dimensionality reduction, but it has the drawback that requires Gaussian distributed data sets; in medical imaging, however, non-Gaussian data sets are frequent^17^. In contrast, nonlinear dimension reduction techniques use the Euclidean distance on a local scale and geodesic distances (Riemann space) on a global scale. This approach enables the representation of these geodesic distances using coordinates within the reduced space. Today, uniform manifold approximation and projection (UMAP)^13^ probably represents the best performing reduction algorithm, more accurately preserving the global structure of the data due to the graph construction correctly modeling the local neighborhood between all individuals. This is the reason why UMAP was selected in our framework to determine the manifold subspace.

In this article, we used manifold learning in a generative manner, allowing us to conserve the usual statistical framework used in medicine for the analysis of individuals, but replacing a global Euclidean framework with a learning manifold based on the set of healthy control individuals selected to globally resemble the test subject under consideration.

Generative manifold learning is an unsupervised machine learning technique, limiting the risk of data leakage recently reported in supervised classification techniques^18^. In addition, the focus of this article is to compare a radiological feature (regional cortical volume) with a pathological feature (hypertrophy). However, as we compare sensitivity in the operated zones, we added a second cohort of 30 supplementary controls, different from the 2944 controls used as reference, to rule out that we are detecting a larger unexpected number of hypertrophies. To note, in our paper the patients were not learned as the manifold learning spaces are unsupervised AI techniques and the residuals were only learned from the controls. Importantly, as it is important to test our personalized normative values in future multicenter studies/external datasets, we purposedly learned the manifold based on controls from various previous studies encompassing multiple MRI manufacturers and sequences; this limits the risk of decreasing the model performance when applied to other datasets. In fact, the lifespan control data used here had been acquired using over one-hundred 1.5 T and 3 T sites.

FCD is a heterogeneous group of cerebral malformations with architectural features of cortical disorganization. They are often responsible for refractory epilepsy and constitute the main cause of surgically-remediable epilepsy in children. Up to 23% of patients receiving surgical resection of seizure foci have an FCD diagnosis ^19^. FCD type II consists of abnormal cortical lamination, specifically, with cytologic abnormalities, either without balloon cells (Type IIa) or with balloon cells (Type IIb) ^20^. FCD radiological diagnosis classically relies on abnormal cortical morphology and/or blurred gray-white matter junction on T1-weighted imaging, therefore making interesting to extract cortical metrics for possible apparent hypertrophy linked to one of these two phenomena.

In 2012, Wang et al.^21^ evoked the potential usefulness of cerebral morphometrics in detecting MRI-negative epilepsies. Jin et al.^22^ tested the performance of neural networks for surface marker-based morphometrics detection of FCD, with an estimated specificity of 90% (by comparison with hippocampal sclerosis) and an estimated sensitivity of 73.7%. The main difference with our work, in addition to the use of 6 cortical features (such as gray-white matter intensity contrast, curvature and sulcal depth), was that Jin et al. proposed a classifier (to differentiate between brains with or without FCD) without localizing the epileptic area. Noteworthy is the more difficult interpretation of surface markers compared to AssemblyNet-based morphometrics, the latter being uniquely based on calculating lesion volume. Interestingly, Chen et al. ^23^ reported that volume-based morphometry analysis can help in detecting FCD lesions individually, but also noted that atrophic regions are more likely than hypertrophic regions to represent FCD lesions. Their study was focused on MRI negative findings from radiologists. We hypothesized that the atrophic regions were misclassified due to the limitation of a Z-score Euclidean framework in the case of non-Gaussian distribution for the region of interest.

More recently, Spitzer et al.^24^ described a multicenter study including 618 patients with FCD and 397 controls, and using a deep-learning algorithm based on 33 morphometrics-derived surface markers. The sensitivity is close to those from our study at 67% despite using 33 metrics (as compared with the unique cortical thickness in our paper) with an estimated specificity of 54%. The sensitivity increased to 85% on a sub-cohort of patients without epileptic seizures and having undergone FLAIR imaging in addition to T1-weighted imaging. Analyses of diagnostic precision fall outside the scope of our study, which instead focused on the resected zone; however, we have checked that our personalized norms are not associated with an increased rate of hypertrophy/atrophy in other cortical zones, as well as when applied to a second cohort of 30 volunteers without any known neurological disorders. Future studies are required to investigate the diagnostic precision of *GeoNorm*.

We note that we are detecting other increases of estimated cortical volume unrelated to patients’ symptoms and that the second group of controls also showed such areas. In epileptic patients, the role of neuroplasticity following seizures could be put forward as a possible reason behind this finding. The presence of cortical areas outside the centile in a second group of 30 controls might be considered as “false positives”. GeoNorm found less outliers than average normative values, although both method succeeds in capturing all cortical variation. In addition, as both models study 132 regions per brain, statistical laws make it possible to detect 5% of regions classified as positive even if they have no abnormality (i.e., decrease and or increase) for a threshold at 5%. A threshold for multiple comparisons is not adapted as the anatomical regions are not studied separately in the reduced subspace.

The use of generative manifold learning as proposed in our article allows a considerable increase in diagnostic performance, since 17 out of 28 patients presented a cortical hypertrophy, compared to 11 out of 28 with the Euclidean statistics model, corresponding to a further 55% more subjects with a detected abnormality. These performances are solely based on T1-weighted imaging and are expected to be increased by adding other complementary information, such as clinical examination, EEG and complementary MRI sequences such as FLAIR.

Interestingly, the manifold learning allowed the detection of 6 abnormalities (versus 2 for the Euclidean model) in patients for whom the MRI was considered normal by the expert radiologist. It should be stressed that all these patients had pathology-confirmed FCD, validating the findings from *GeoNorm*. Future prospective and multicenter studies including EEG and clinical data will permit an evaluation of the potential value of these new personalized normative values for the detection of FCDII, ideally included in a clinical decision support system^25^.

The results found in the supplementary Alzheimer’s disease cohort hold promise for earlier detection of Alzheimer’s Disease, as *GeoNorm* described more hippocampus injuries in both pMCI and AD groups, suggesting increased sensitivity. We also found 8 cognitively normal controls with decreased hippocampus volume, while average normative values only found 2 controls with such feature, without statistical significative difference between the two groups. In our opinion, 2 out of 155 (as detected by the average normative values) is a very low rate in an elder population of controls because radiological signs of AD may occur decades before clinical signs, and regarding the high prevalence of AD in this population. Of note, however, in contrast to the focal cortical dysplasia cohort (main cohort in this study), there was no gold standard data regarding the presence or absence of hippocampal abnormality.

In conclusion, we propose *GeoNorm* as a new manifold learning technique to definine personalized normative values for quantitative MRI and more generally quantitative medicine. The possibility to define a ‘digital twin’ and adapt the reference values to both intra- and inter-subjects’ variability holds great promise for precision medicine.

## Methods

### Ethics approval

All datasets were collected under Institutional Review Board (IRB) approval from the French Society of Radiology (Ethic committee of research) with the number CRM-2302-320. The datasets were de-identified prior to model development. Due to the retrospective nature of the study, the IRB from the French Society of Radiology (https://cerf.radiologie.fr/cerim) waived the need of obtaining informed consent. All experiments were performed in accordance with relevant guidelines and regulations, including the Declaration of Helsinki.

### Subjects

#### Epilepsy patient selection

Adult and pediatric patients were retrospectively identified from consecutive patients at our institution who underwent resective surgery for epilepsy between 2011 and 2022 and had FCD type II confirmed by post-resection pathology. All patients underwent video–scalp EEG (SEEG) long-term monitoring and brain MRI. Noninvasive data were presented at local epilepsy conferences to provide a consensual conclusion regarding the most likely epileptogenic zone and the decision to proceed directly to surgery or to perform SEEG procedure for patients with MRI-negative patients.

Similar to the decision on whether to proceed to SEEG, the type and extent of surgical treatment was discussed and approved at local epilepsy conferences based on review of available data.

FCD pathology was classified as type I, IIA, IIB, or III according to the International League Against Epilepsy 2010 classification. Initially, we found 82 eligible patients who underwent a cortectomy in our center with a FCD type II on the pathological analysis. Fifty-four patients were excluded because of the lack of available presurgical MRI. We thus retrospectively included 28 patients who met the inclusion criteria. Among these patients, 12 were women and 16 men. Most of them were right-handed (n = 23; 82%). Average onset age of epileptic crisis was 10.2 years [0.5–39]. Eleven patients had neurological, familial or personal history and predispositions, and four among them had familial history of epilepsy. All 28 patients included had a presurgical MRI at mean age of 24.1 years [1–54 years]. The MRI data were acquired on different scanners (1.5 Tesla: n = 13; 3 Tesla: n = 15). Seventeen patients (60.7%) were considered abnormal by an expert neuroradiologist. The histologically proved FCDII were localized in the frontal lobe (n = 19, including 3 in the supplementary motor areas), temporal lobe (n = 5), parietal lobe (n = 3) and occipital lobe (n = 1). All these characteristics are described in Table 1.

#### Healthy control datasets

We used 3D T1-weighted MRI data from nine publicly available databases covering the entire lifespan (see “Acknowledgments ”section). All subjects included are normal controls. The images were acquired at 1.5 T and 3 T over 103 sites. From the initial 3,296 subjects, and after assessing for quality control, 2,944 subjects (47% female; 9 months to 94 years, with an average age of 39.7 years and standard deviation of 26.6 years) were kept. The Alzheimer’s Disease Neuroimaging Initiative (ADNI) data, including cognitively normal and Alzheimer’s Disease patients, were only used in the external validation testing (see supplementary material). The details of the dataset have been provided^5^.

For comparison purpose with the findings from the 28 epileptic patients, we also included 30 supplementary healthy subjects locally scanned in our academic hospital, which were never learnt in the initial manifold. These subjects are matched in gender and age with the epileptic patients. The 3D T1-weighted MRI data for this group were acquired on different scanners, on a Siemens Aera 1.5 Tesla (n = 16) and on two 3 Tesla MR machine (Philips Ingenia, n = 9 and Siemens Skyra, n = 5).

### Framework

#### AssemblyNet

All the considered T1-weighted MRI data were segmented using AssemblyNet (https://github.com/volBrain/AssemblyNet/). This software produces a deep learning based segmentation (i.e., 132 structures) of the entire brain as part of the volBrain pipeline^1^ with the following steps: (i) denoising^27^, (ii) inhomogeneity correction^28^, (iii) affine registration into the MNI space^29^, tissue-based intensity normalization^30^, and (v) intracranial cavity volume (ICV) extraction^31^. Finally, image intensities were centralized and normalized within the brain mask and the background was set to zero. After preprocessing, the brain was automatically segmented into 132 structures using 250 Deep Learning models (see supplementary table 2)^1^.

#### Lifespan model

To compensate for the inter-subject variability, we normalized all the structure volumes using the ICV. The lifespan model of the different structure volumes is modeled using low order polynomial regression related to age on the normal control datasets as described in a previous publication of Coupé et al. ^5^. The normative range/interval is estimated using a threshold at 5% for the lower bound and 95% for the upper bound.

#### GeoNorm

**Figure 3:**
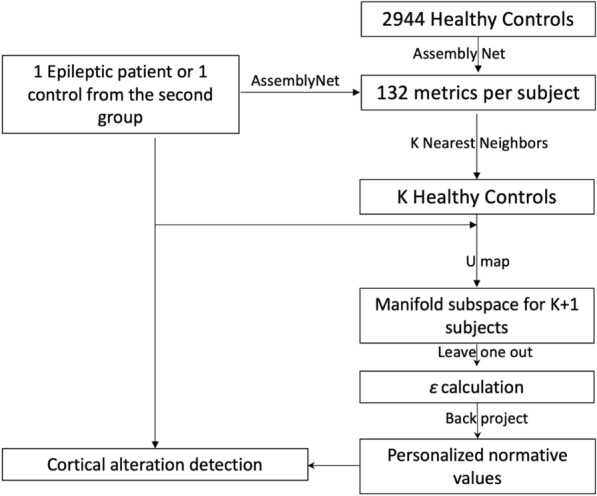
Pipeline in the proposed GeoNorm framework. For each subject of interest (e.g. an epileptic patient), the K-nearest neighbors from the large healthy control group are identified (in this study, K = 30). The nearest controls, together with the subject of interest were moved to a reduced manifold subspace (which was computed from the 30 nearest controls). We considered that the residuals ε was representative of any abnormalities present in a new patient when it is greater than the model variability learned during a leave-one-out procedure on controls.

GeoNorm are part of the BrainGML software (version 0.1,) a medical device software that assist radiologic assessment of patients with neurological disorders We used a strategy similar to that developed in *TractLearn*^15^ to generate the personalized normative values (see Fig. 3 for a summary of the proposed pipeline). We first estimated the distance between a given epileptic patient and the 30 closest controls from the full 2,944 set in our normal control dataset. To identify this closest group (for each patient), we used volume differences between normalized volumes for each brain structure of the epileptic patient and selected the 30 controls with the shortest distances. For each of the epileptic patients and the subjects from the second control dataset, we reduced the 132 quantitative variables coming from the *AssemblyNet* into a low-dimensional reduced manifold space using U-map^13^ for the 31-subject set (i.e., the subject of interest + the 30 closest healthy controls). In practice, U-map was therefore used 28 times for the epileptic patient group and 30 times for the second dataset of controls.

We learn the manifold subspaces based on the data from the healthy controls:

$$Y \, = \, f\left( x \right) + \varepsilon$$

where *Y* is the healthy control data in real space (i.e., the 132 quantitative values extracted from AssemblyNet), *x* the corresponding point in the reduced space, and *ε* the residuals; *f* is the regression function between the reduced space and the real space. The combined manifold learning algorithm and regression functions determine the quality of the model. The regression function is crucial to obtain the smallest residual; if it cannot capture the variation estimated between the real and the latent space, the residual will be high.

The projection *f* of a new brain (i.e. not included in the learned manifold step) will be based on the collection of brain measures from the closest subjects from the healthy manifold. As the Riemannian normative space has been built to capture the maximum variability, a new subject projected onto the learned manifold will be synthetized using a *local* average from the closest 30 healthy controls. For the estimation of the local average, we have used the Nadaraya-Watson kernel for high dimensional non-parametric regression^32^, here defined as:

$$\hat{Y} = \frac{{\mathop \sum \nolimits_{i} K_{h} \left( {x - x_{i} } \right)y_{i} }}{{\mathop \sum \nolimits_{j} K_{h} \left( {x - x_{j} } \right)}}$$

where *K*_*h*_ represents a Gaussian kernel of bandwidth *h*, which was selected to reduce the residuals between *Y* and *Ŷ*. The training couples (*x*_*i*_*, y*_*i*_) includes *x* as the quantity to regress and *y* as the prediction.

Two toy examples illustrating the differences between average normative values and personalized normative values are provided in Figs. S1 and S2.

### Statistical analysis

To ensure robustness in our dataset and avoid overfitting, the distribution of the residuals ε of the healthy group was estimated using a leave-one-out (LOO) strategy. The subjects used to estimate the model (the latent space and the regression function) are not used in the estimation of the parameters of the distribution law of the residuals. We consider that the residual ε of a new subject is representative of the abnormalities present in that subject when it is greater than the model variability learned during the LOO on the healthy control group. Permutation tests were computed for the 30 nearest neighbors. We assume that ε follows a multivariate Gaussian distribution with a standard deviation that varies across the voxels^33,34^.

Following the LOO strategy, we obtained the distribution of the residuals of the control group. We considered the mean of this last distribution to estimate the z-score. We can also evaluate the confidence interval of the residual of the new subject at the N-th percentage level, to evaluate the robustness of the method.

While we benefited from the information provided by a surgical gold standard for the epileptic patients, we still need to confirm that we are not finding an unexpected amount of decreased/increased estimated cortical volume in normal brain cortical areas in patients or in our second cohort of 30 supplementary healthy controls. We hypothesize that the second cohort of controls would detect a lower number of atypical regions than those of epileptic patients with both models (average normative values and the personalized normative values). We have then compared the *GeoNorm* framework with the lifespan model in all brain regions for 58 individual subjects (28 epileptic patients and 30 controls) to detect subject-specific atrophy and hypertrophy using a Wilcoxon rank pair test. We also looked for a potential disagreement between the performance of the average normative values and the personalized normative values with a Mc Nemar test.

### Supplementary Information

Supplementary Information.

## Data Availability

The C-MIND data used in the preparation of this article were obtained from the C-MIND Data Repository (accessed in February 2015) created by the C-MIND study of Normal Brain Development. This is a multisite, longitudinal study of typically developing children from ages newborn through young adulthood conducted by Cincinnati Children’s Hospital Medical Center and UCLA A listing of the participating sites and a complete listing of the study investigators can be found at https://research.cchmc.org/c-mind. The NDAR data used in the preparation of this manuscript were obtained from the NIH-supported National Database for Autism Research (NDAR). NDAR is a collaborative informatics system created by the National Institutes of Health to provide a national resource to support and accelerate research in autism. The NDAR dataset includes data from the NIH Pediatric MRI Data Repository created by the NIH MRI Study of Normal Brain Development. This is a multisite, longitudinal study of typically developing children from ages newborn through young adulthood conducted by the Brain Development Cooperative Group A listing of the participating sites and a complete listing of the study investigators can be found at http://pediatricmri.nih.gov/nihpd/info/participating_centers.html. The ADNI data used in the preparation of this manuscript were obtained from the Alzheimer’s Disease Neuro- imaging Initiative (ADNI). The ADNI is funded by the National Institute on Aging and the National Institute of Biomedical Imaging and Bioengineering and through generous contributions from the following: Abbott, AstraZeneca AB, Bayer Schering Pharma AG, Bristol-Myers Squibb, Eisai Global Clinical Development, Elan Corporation, Genentech, GE Healthcare, GlaxoSmithKline, Innogenetics NV, Johnson & Johnson, Eli Lilly and Co., Medpace, Inc., Merck and Co., Inc., Novartis AG, Pfizer Inc., F. Hoffmann-La Roche, Schering-Plough, Synarc Inc., as well as nonprofit partners, the Alzheimer’s Association and Alzheimer’s Drug Discovery Foundation, with participation from the U.S. Food and Drug Administration. Private sector contributions to the ADNI are facilitated by the Foundation for the National Institutes of Health (www.fnih.org). The grantee organization is the Northern California Institute for Research and Education, and the study was coordinated by the Alzheimer’s Disease Cooperative. Study at the University of California, San Diego. ADNI data are disseminated by the Laboratory for NeuroImaging at the University of California, Los Angeles. The OASIS data used in the preparation of this manuscript were obtained from the OASIS project. See http://www.oasis-brains.org/ for more details. The AIBL data used in the preparation of this manuscript were obtained from the AIBL study of ageing. See www.aibl.csiro.au for further details. The ICBM data used in the preparation of this manuscript. The IXI data used in the preparation of this manuscript were supported by http://www.brain-development.org/. The ABIDE data used in the preparation of this manuscript were supported by ABIDE funding resources listed at http://fcon_1000.projects.nitrc.org/indi/abide/preprocessed.html. ABIDE primary support for the work by Adriana Di Martino. Primary support for the work by Michael P. Milham and the INDI team was provided by gifts from Joseph P. Healy and the Stavros Niarchos Foundation to the Child Mind Institute. http://fcon_1000.projects.nitrc.org/indi/abide/preprocessed.html. This manuscript reflects the views of the authors and may not reflect the opinions or views of the database providers. The dataset for the epileptic patients is available from the corresponding author on reasonable request.

## Code availability

The underlying code for AssemblyNet can be accessed via this link: https://github.com/volBrain/AssemblyNet. The code for BrainGML is not publicly available for proprietary reasons.
