# Supplementary material for: Data-driven normative values based on generative manifold learning for quantitative MRI
Source: Sci Rep. 2024 Mar 30;14:7563. doi: 10.1038/s41598-024-58141-4 (PMC10981723; doi:10.1038/s41598-024-58141-4)
Supplement: Supplementary file 1 — Supplementary Information. [file 41598_2024_58141_MOESM1_ESM.docx]

**Supplementary material**


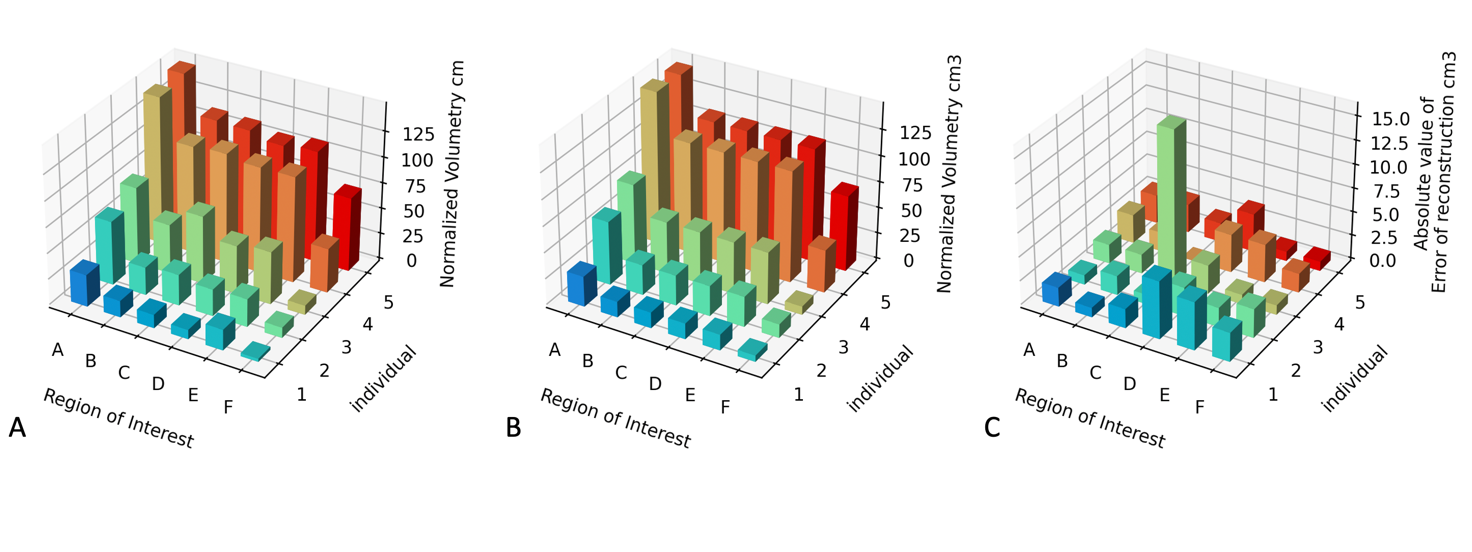


*Figure S1: Toy example of 5 subjects with 6 different regions of interest (ROIs). Each subject is represented by a single color. The height of the bar plot corresponds to the normalized volumetry. In this toy example, the subjects are linked (i.e. subjects can also differ by an overall scaling, globally affecting the quantitative values for all ROIs). Subject 3 had cortex hyperplasia in ROI C. Figure 5A (which displays the real data Y) shows for the ROI C row that subject 3 does not appear as an outlier in comparison with the 4 other subjects. Figure 5B represents the digital twin (i.e. the model Ŷ=f(x)) for all 5 subjects (build based on all cortical metrics), showing a visual difference for ROI C of subject 3 when compared with Figure 5A. The last part (Figure 5C) highlights the error of reconstruction (i.e. the absolute value of the residual ε), which is larger for ROI C of subject 3 than for all other subjects and areas.*


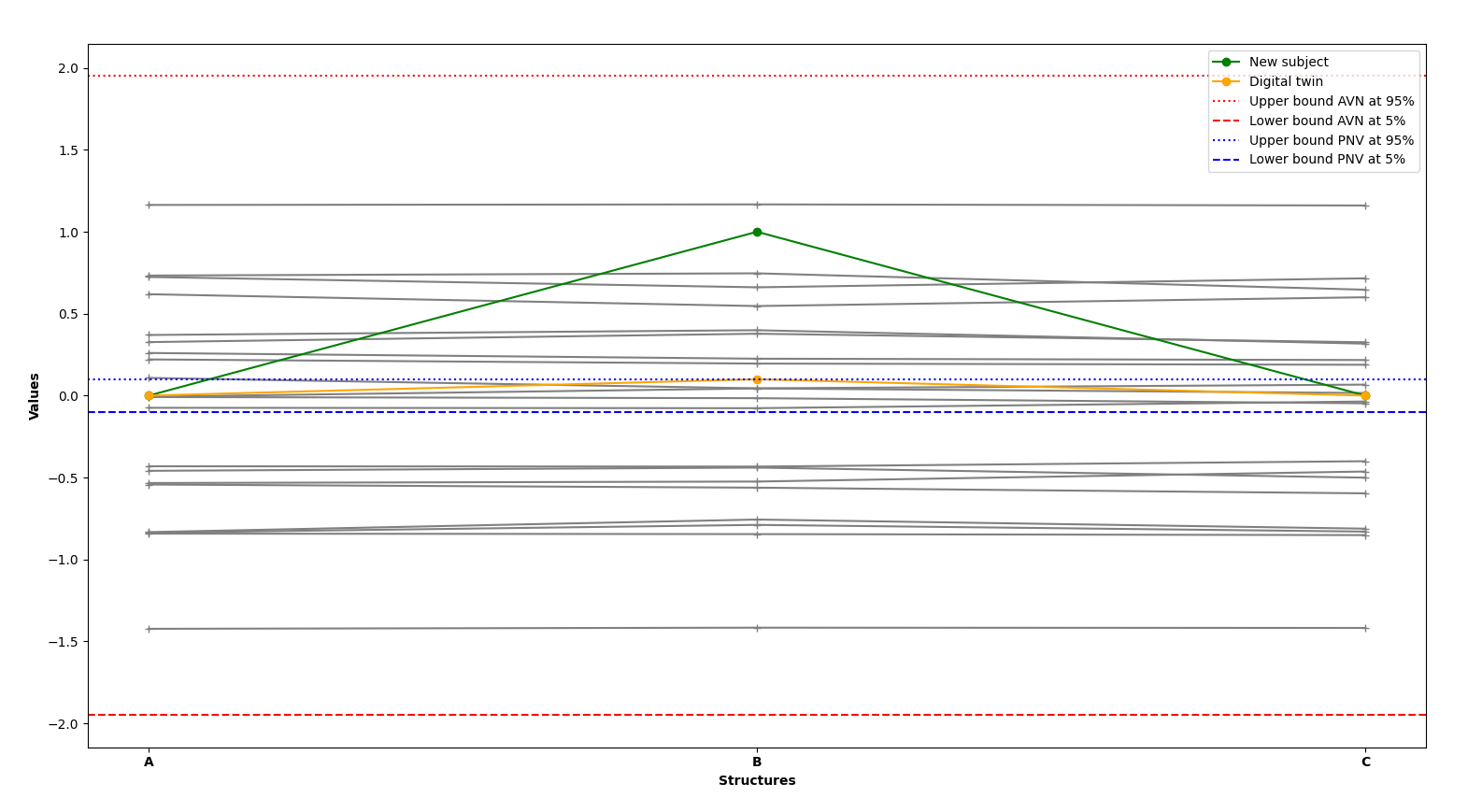


*Figure S2: Average normative values (ANV, represented as dotted red lines) and personalized normative values (PNV, represented as blue lines red lines) calculated from a cohort of 20 normal controls (grey lines) for 3 random structures of the brain. The values represent the typical interval for Z-Scores variation, being significant between +1.96 and -1.96. In the case of a new subject (green line) with atypical variation of the second structure Z-score value in comparison with A and C structures Z-scores, the lower and upper ANR fail to detect B structure as an outlier, while PNR calculated from the digital twin (yellow line) accurately capture the B structure as outlier.*

*Supplementary Table 1. Comparison of average normative values (traditional LifeSpan method) and personalized normative values (proposed GeoNorm method) for evaluation of hippocampi atrophy in Alzheimer’ Disease (AD) and progressive Mild Cognitive Impairment (pMCI) models. For the purpose of this comparison, we used exactly the same framework on controls coming from the ADNI dataset (n=155), pMCI patients (n=165) and AD patients (n=332). All subjects which pass the quality control of AssemblyNet were included.*

|  | Average normative values  (LifeSpan) | Personalized normative values  (GeoNorm) |
| --- | --- | --- |
| Number of AD patients with hippocampus volume decrease | 214 out of 332 | 248 out of 332 |
| Number of pMCI with hippocampus volume decrease | 90 out of 165 | 110 out of 165 |
| Number of controls with hippocampus volume decrease | 2 out of 155 | 8 out of 155 |

Personalized normative values significantly detected more hippocampus atrophy in comparison with average normative values in the pMCI group (p= 0.024) and in the AD group (p=0.009) using the Wilcoxon tests. There was no significant difference in the control group using the same test (p=0.107).

Supplementary Table 2: List of the 133 labels (132 structures and the background) segmented by AssemblyNet.

| # | Label Number | Structure name |
| --- | --- | --- |
| 1 | 0 | background |
| 2 | 4 | 3rd-Ventricle |
| 3 | 11 | 4th-Ventricle |
| 4 | 23 | Right Accumbens |
| 5 | 30 | Left Accumbens |
| 6 | 31 | Right Amygdala |
| 7 | 32 | Left Amygdala |
| 8 | 35 | Brain Stem |
| 9 | 36 | Right Caudate |
| 10 | 37 | Left Caudate |
| 11 | 38 | Right Cerebellum Exterior |
| 12 | 39 | Left Cerebellum Exterior |
| 13 | 40 | Right Cerebellum White Matter |
| 14 | 41 | Left Cerebellum White Matter |
| 15 | 44 | Right Cerebral White Matter |
| 16 | 45 | Left Cerebral White Matter |
| 17 | 47 | Right Hippocampus |
| 18 | 48 | Left Hippocampus |
| 19 | 49 | Right Inf Lat Ventricle |
| 20 | 50 | Left Inf Lat Ventricle |
| 21 | 51 | Right Lateral Ventricle |
| 22 | 52 | Left Lateral Ventricle |
| 23 | 55 | Right Pallidum |
| 24 | 56 | Left Pallidum |
| 25 | 57 | Right Putamen |
| 26 | 58 | Left Putamen |
| 27 | 59 | Right Thalamus |
| 28 | 60 | Left Thalamus |
| 29 | 61 | Right Ventral DC |
| 30 | 62 | Left Ventral DC |
| 31 | 71 | Cerebellar Vermal Lobules-I-V |
| 32 | 72 | Cerebellar Vermal Lobules-VI-VII |
| 33 | 73 | Cerebellar Vermal Lobules-VIII-X |
| 34 | 75 | Left Basal Forebrain |
| 35 | 76 | Right Basal Forebrain |
| 36 | 100 | Right anterior-cingulate-gyrus |
| 37 | 101 | Left anterior-cingulate-gyrus |
| 38 | 102 | Right anterior-insula |
| 39 | 103 | Left anterior-insula |
| 40 | 104 | Right anterior-orbital-gyrus |
| 41 | 105 | Left anterior-orbital-gyrus |
| 42 | 106 | Right angular-gyrus |
| 43 | 107 | Left angular-gyrus |
| 44 | 108 | Right calcarine-cortex |
| 45 | 109 | Left calcarine-cortex |
| 46 | 112 | Right central-operculum |
| 47 | 113 | Left central-operculum |
| 48 | 114 | Right cuneus |
| 49 | 115 | Left-cuneus |
| 50 | 116 | Right entorhinal-area |
| 51 | 117 | Left entorhinal-area |
| 52 | 118 | Right frontal-operculum |
| 53 | 119 | Left frontal-operculum |
| 54 | 120 | Right frontal-pole |
| 55 | 121 | Left frontal-pole |
| 56 | 122 | Right fusiform-gyrus |
| 57 | 123 | Left fusiform-gyrus |
| 58 | 124 | Right gyrus-rectus |
| 59 | 125 | Left gyrus-rectus |
| 60 | 128 | Right inferior-occipital-gyrus |
| 61 | 129 | Left inferior-occipital-gyrus |
| 62 | 132 | Right inferior-temporal-gyrus |
| 63 | 133 | Left inferior-temporal-gyrus |
| 64 | 134 | Right lingual-gyrus |
| 65 | 135 | Left lingual-gyrus |
| 66 | 136 | Right lateral-orbital-gyrus |
| 67 | 137 | Left lateral-orbital-gyrus |
| 68 | 138 | Right middle-cingulate-gyrus |
| 69 | 139 | Left middle-cingulate-gyrus |
| 70 | 140 | Right medial-frontal-cortex |
| 71 | 141 | Left medial-frontal-cortex |
| 72 | 142 | Right middle-frontal-gyrus |
| 73 | 143 | Left middle-frontal-gyrus |
| 74 | 144 | Right middle-occipital-gyrus |
| 75 | 145 | Left middle-occipital-gyrus |
| 76 | 146 | Right medial-orbital-gyrus |
| 77 | 147 | Left medial-orbital-gyrus |
| 78 | 148 | Right postcentral-gyrus |
| 79 | 149 | Left postcentral-gyrus |
| 80 | 150 | Right precentral-gyrus |
| 81 | 151 | Left precentral-gyrus |
| 82 | 152 | Right superior-frontal-gyrus |
| 83 | 153 | Left superior-frontal-gyrus |
| 84 | 154 | Right middle-temporal-gyrus |
| 85 | 155 | Left middle-temporal-gyrus |
| 86 | 156 | Right occipital-pole |
| 87 | 157 | Left occipital-pole |
| 88 | 160 | Right occipital-fusiform-gyrus |
| 89 | 161 | Left occipital-fusiform-gyrus |
| 90 | 162 | Right opercular inf. frontal gyrus |
| 91 | 163 | Left opercular inf. frontal gyrus |
| 92 | 164 | Right orbital inf. frontal gyrus |
| 93 | 165 | Left orbital inf. frontal gyrus |
| 94 | 166 | Right posterior-cingulate-gyrus |
| 95 | 167 | Left posterior-cingulate-gyrus |
| 96 | 168 | Right precuneus |
| 97 | 169 | Lef precuneus |
| 98 | 170 | Right parahippocampal-gyrus |
| 99 | 171 | Lef parahippocampal-gyrus |
| 100 | 172 | Right posterior-insula |
| 101 | 173 | Left posterior-insula |
| 102 | 174 | Right parietal-operculum |
| 103 | 175 | Left parietal-operculum |
| 104 | 176 | Right postcentral-gyrus |
| 105 | 177 | Left postcentral gyrus |
| 106 | 178 | Right posterior orbital gyrus |
| 107 | 179 | Left posterior orbital gyrus |
| 108 | 180 | Right planum polare |
| 109 | 181 | Left planum polare |
| 110 | 182 | Right precentral gyrus |
| 111 | 183 | Left precentral gyrus |
| 112 | 184 | Right planum temporale |
| 113 | 185 | Left planum temporale |
| 114 | 186 | Right subcallosal area |
| 115 | 187 | Left subcallosal area |
| 116 | 190 | Right sup. frontal gyrus |
| 117 | 191 | Left sup. frontal gyrus |
| 118 | 192 | Right supplementary motor cortex |
| 119 | 193 | Left supplementary motor cortex |
| 120 | 194 | Right supramarginal gyrus |
| 121 | 195 | Left supramarginal gyrus |
| 122 | 196 | Right sup. occipital gyrus |
| 123 | 197 | Left sup. occipital gyrus |
| 124 | 198 | Right sup. parietal lobule |
| 125 | 199 | Left sup. parietal lobule |
| 126 | 200 | Right sup. temporal gyrus |
| 127 | 201 | Left sup. temporal gyrus |
| 128 | 202 | Right temporal pole |
| 129 | 203 | Left temporal pole |
| 130 | 204 | Right triangular inf. frontal gyrus |
| 131 | 205 | Left triangular inf. frontal gyrus |
| 132 | 206 | Right transverse temporal gyrus |
| 133 | 207 | Left transverse temporal gyrus |
